# Supplementary material for: Super-resolution expansion microscopy reveals nanoscale protein domains and CO2-dependent remodeling of Chlamydomonas pyrenoid-traversing membranes
Source: bioRxiv. 2026 Jul 7:2026.06.11.731689. Preprint. [Version 2] doi: 10.64898/2026.06.11.731689 (PMC13370406; doi:10.64898/2026.06.11.731689)
Supplement: Supplement 1 [file NIHPP2026.06.11.731689v2-supplement-1.pdf]

bioRxiv preprint doi: https://doi.org/10.64898/2026.06.11.731689; this version posted July 7, 2026. The copyright holder for this preprint (which was not certified by peer review) is the author/funder, who has granted bioRxiv a license to display the preprint in perpetuity. It is made available under a CC-BY 4.0 International license.

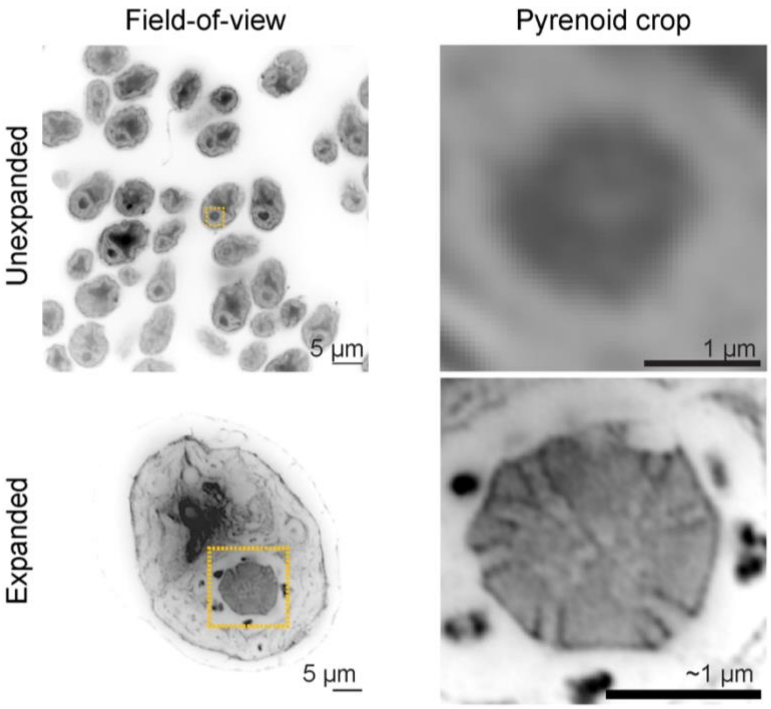

Figure S1: Additional examples of unexpanded and expanded Chlamydomonas cells grown for 6 hours at low CO_2.

bioRxiv preprint doi: https://doi.org/10.64898/2026.06.11.731689; this version posted July 7, 2026. The copyright holder for this preprint (which was not certified by peer review) is the author/funder, who has granted bioRxiv a license to display the preprint in perpetuity. It is made available under a CC-BY 4.0 International license.

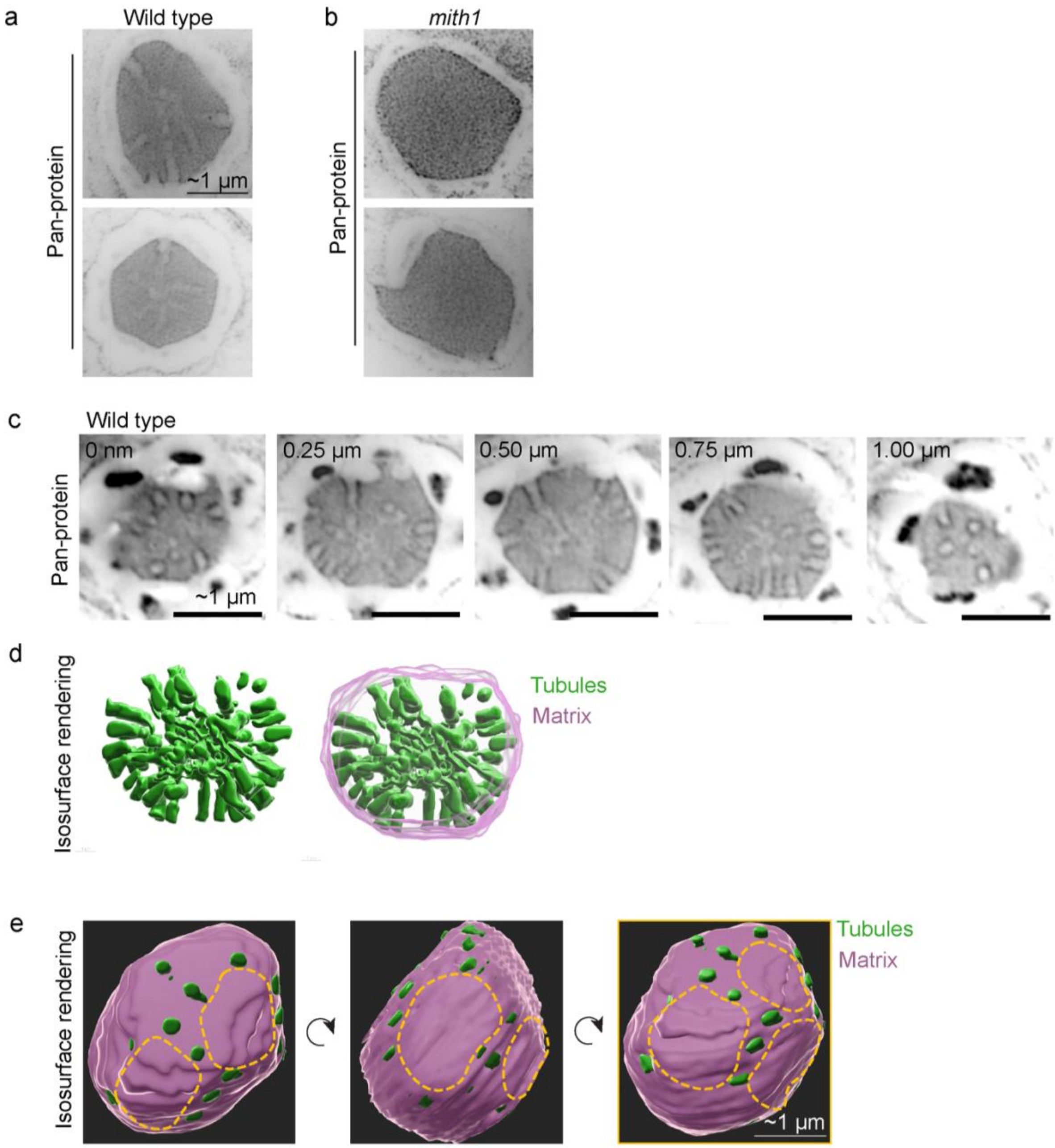

Figure S2: Additional examples of wild-type and *mith1* mutant pyrenoids and wild-type pyrenoid space-filling renderings.

a. Additional examples of medial slices of wild-type pyrenoids stained with pan-protein stain. **b.** Additional examples of medial slices of *mith1* pyrenoids stained with pan-protein stain. **c.** Confocal z-series of the wild-type pyrenoid from **Figure S1** stained with

bioRxiv preprint doi: https://doi.org/10.64898/2026.06.11.731689; this version posted July 7, 2026. The copyright holder for this preprint (which was not certified by peer review) is the author/funder, who has granted bioRxiv a license to display the preprint in perpetuity. It is made available under a CC-BY 4.0 International license.

pan-protein stain. d. Space-filling reconstructions of tubules and matrix segmented from the confocal sections in **c**. **e.** Oblique views of the space-filling rendering in **Figure 2c**, with the dotted lines representing possible locations of starch granules between the linear arrangements of tubules. Scale bars corrected for expansion factor.

bioRxiv preprint doi: https://doi.org/10.64898/2026.06.11.731689; this version posted July 7, 2026. The copyright holder for this preprint (which was not certified by peer review) is the author/funder, who has granted bioRxiv a license to display the preprint in perpetuity. It is made available under a CC-BY 4.0 International license.

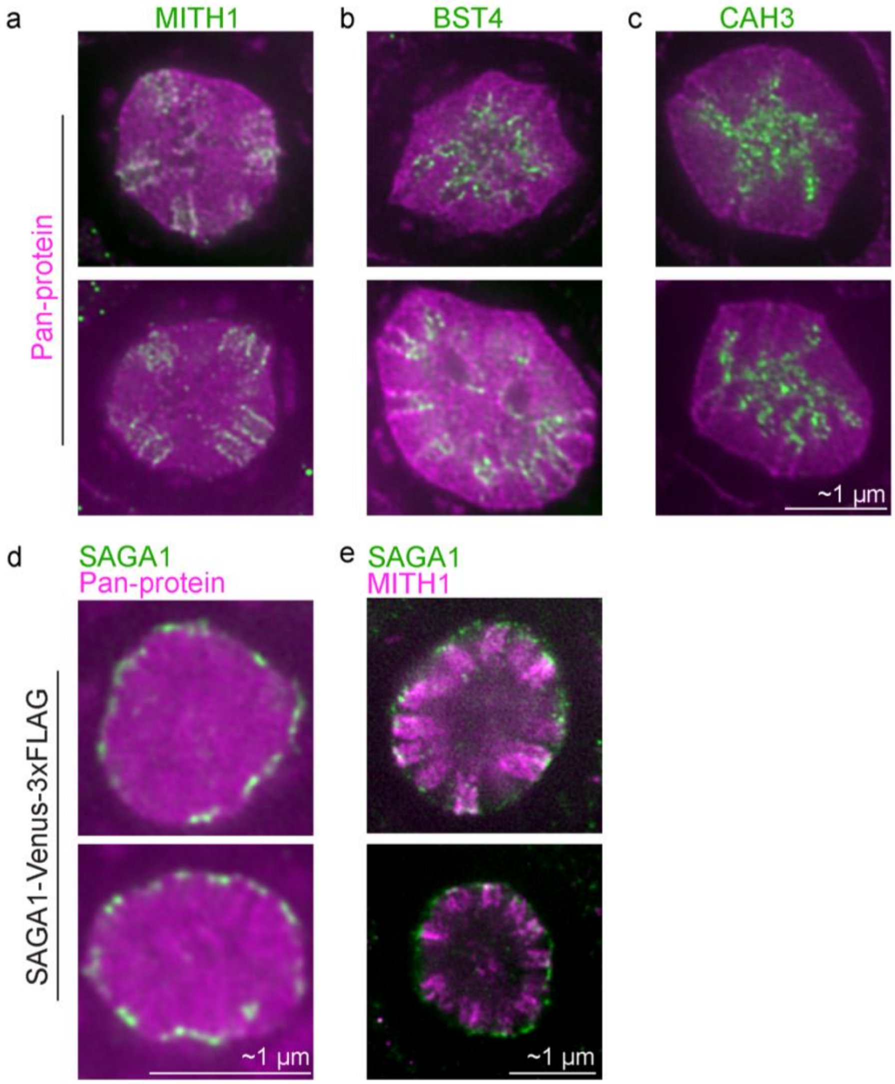

Figure S3: Additional examples of MITH1, BST4, CAH3 and SAGA1 immunofluorescence localization.

a-c. Wild-type pyrenoids displayed with immunofluorescence (green) merged with pan-protein stain (magenta). The antibodies used were a. MITH1, b. BST4, and c. CAH3.

bioRxiv preprint doi: https://doi.org/10.64898/2026.06.11.731689; this version posted July 7, 2026. The copyright holder for this preprint (which was not certified by peer review) is the author/funder, who has granted bioRxiv a license to display the preprint in perpetuity. It is made available under a CC-BY 4.0 International license.

d-e. SAGA1-Venus-3xFLAG expressing pyrenoids displayed with **d.** FLAG (green) and pan-protein stain (magenta) and **e.** FLAG (green) and MITH1 (magenta). Medial slices are displayed for each pyrenoid. Scale bars are corrected for expansion factor.

bioRxiv preprint doi: https://doi.org/10.64898/2026.06.11.731689; this version posted July 7, 2026. The copyright holder for this preprint (which was not certified by peer review) is the author/funder, who has granted bioRxiv a license to display the preprint in perpetuity. It is made available under a CC-BY 4.0 International license.

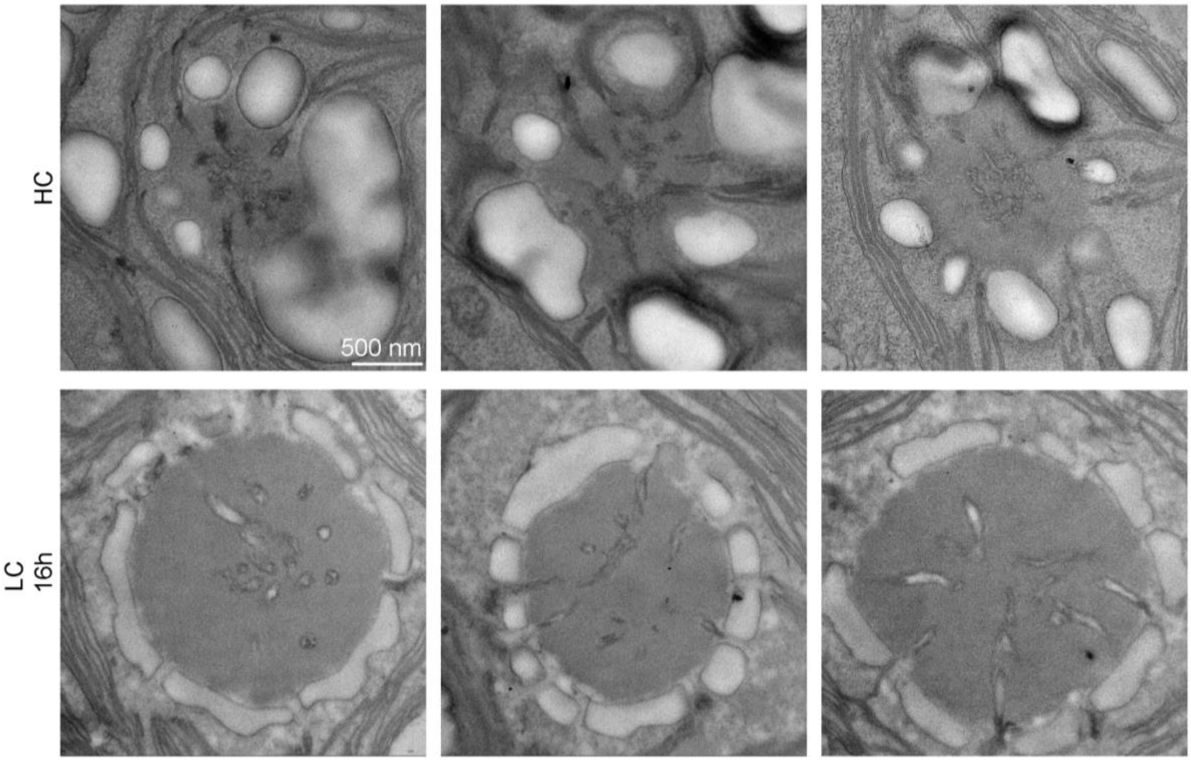

Figure S4: Additional examples of transmission electron micrographs of pyrenoids at high CO_2 and low CO_2.

Transmission electron micrographs (TEM) of wild-type pyrenoids from cells grown at HC and LC 16h. Scale bar 500 nm.

Movie S1: U-ExM + iSIM confocal Z-series of a wild-type pyrenoid stained with pan-protein stain

Scale bar ~1 μm, corrected for expansion factor. Slices every ~33 nm, corrected for expansion factor.

Movie S2: Space-filling rendering of a wild-type pyrenoid matrix (gray) and tubule network (green).

bioRxiv preprint doi: https://doi.org/10.64898/2026.06.11.731689; this version posted July 7, 2026. The copyright holder for this preprint (which was not certified by peer review) is the author/funder, who has granted bioRxiv a license to display the preprint in perpetuity. It is made available under a CC-BY 4.0 International license.

Movie S3: Montage of confocal Z-series of wild-type pyrenoids combining immunofluorescence (green) with pan-protein stain (magenta).
The antibodies used were **a.** MITH1, **b.** BST4 and **c.** CAH3. Scale bars ~1 µm, expansion corrected.

Movie S4: Confocal Z-series of the pyrenoid of SAGA1-Venus-3xFLAG-expressing cell combining anti-FLAG (green) and anti-MITH1 (magenta) immunofluorescence.
Scale bar ~1 µm, expansion corrected.

Table S1: List of Chlamydomonas strains used in this study along with their sources.

Chlamydomonas Resource Center ID	Strain description	Source	Antibiotic resistance
CC-4533	CMJ030	Wild type and parent strain to CLiP1 library	none
LMJ.RY0402.133670	*mith1*	CLiP1 library LMJ.RY0402.133670	Paromomycin
CC-5422	*saga1*; SAGA1-Venus-3xFLAG	Previously published[46]	Paromomycin, Hygromycin
